# Risk Factors for Poor Outcomes of Diabetes Patients With COVID-19: A Single-Center, Retrospective Study in Early Outbreak in China

**DOI:** 10.3389/fendo.2020.571037

**Published:** 2020-09-24

**Authors:** Nan Zhang, Cheng Wang, Feng Zhu, Hong Mao, Peng Bai, Lu-Lu Chen, Tianshu Zeng, Miao-Miao Peng, Kang Li Qiu, Yixuan Wang, Muqing Yu, Shuyun Xu, Jianping Zhao, Na Li, Min Zhou

**Affiliations:** ^1^Department of Endocrinology, Union Hospital, Tongji Medical College, Huazhong University of Science and Technology, Wuhan, China; ^2^Department of Cardiology, Union Hospital, Tongji Medical College, Huazhong University of Science and Technology, Wuhan, China; ^3^Department of Endocrinology, Tongji Medical College, Central Hospital of Wuhan, Huazhong University of Science and Technology, Wuhan, China; ^4^Department of Cardiovascular Surgery, Union Hospital, Tongji Medical College, Huazhong University of Science and Technology, Wuhan, China; ^5^Department of Pulmonary and Critical Care Medicine, Tongji Hospital, Tongji Medical College, Huazhong University of Science and Technology, Wuhan, China; ^6^Key Laboratory of Respiratory Diseases, National Ministry of Health of the People's Republic of China and National Clinical Research Center for Respiratory Disease, Wuhan, China

**Keywords:** SARS-CoV-2, COVID-19, Diabetes Mellitus, risk factors, severe clinical events

## Abstract

**Background:** Diabetes has been found to increase severity and mortality under the current pandemic of coronavirus disease of 2019 (COVID-19). Up to date, the clinical characteristics of diabetes patients with COVID-19 and the risk factors for poor clinical outcomes are not clearly understood.

**Methods:** The study was retrospectively carried out on enrolled diabetes patients with laboratory confirmed COVID-19 infection from a designated medical center for COVID-19 from January 25th, 2020 to February 14th, 2020 in Wuhan, China. The medical record was collected and reviewed. Univariate and multivariate analyses were performed to assess the risk factors associated with the severe events which were defined as a composite endpoint of admission to intensive care unit, the use of mechanical ventilation, or death.

**Results:** A total of 52 diabetes patients with COVID-19 were finally included in the study. 21 (40.4%) patients had developed severe events in 27.50 (IQR 12.25–35.75) days follow-up, 15 (28.8%) patients experienced life-threatening complications and 8 patients died with a recorded mortality rate of 15.4%. Only 13 patients (41.9%) were in optimal glycemic control with HbA1c value of <7.0%. In addition to general clinical characteristics of COVID-19, the severe events diabetes patients showed higher counts of white blood cells and neutrophil, lower lymphocytes (40, 76.9%), high levels of hs-CRP, erythrocyte sedimentation rate (ESR) and procalcitonin (PCT) as compared to the non-severe diabetes patients. Mild higher level of cardiac troponin I (cTNI) (32.0 pg/ml; IQR 16.80–55.00) and D-dimer (1.70 μg/L, IQR 0.70–2.40) were found in diabetes patients with severe events as compared to the non-severe patients (cTNI:20.00 pg/ml, IQR5.38–30.00, *p* = 0.019; D-dimer: 0.70 μg/L, IQR 0.30–2.40, *p* = 0.037). After adjusting age and sex, increased level of cTNI was found to significantly associate with the incidence of severe events (HR: 1.007; 95% CI: 1.000–1.013; *p* = 0.048), Furthermore, using of α-glucosidase inhibitors was found to be the potential protectant for severe events (HR: 0.227; 95% CI: 0.057–0.904; *p* = 0.035).

**Conclusion:** Diabetes patients with COVID-19 showed poor clinical outcomes. Vigorous monitoring of cTNI should be recommended for the diabetes patients with COVID-19. Usage of α-glucosidase inhibitors could be a potential protectant for the diabetes patients with COVID-19.

## Introduction

Since late December 2019, a number of pneumonia cases caused by a novel coronavirus named Severe Acute Respiratory Syndrome Coronavirus 2 (SARS-CoV-2) has been reported in Wuhan, China. The resultant disease from SARS-CoV-2 infection is named as Coronavirus Disease of 2019 (COVID-19) by the World Health Organization (WHO) (1). The COVID-19 pandemic has so far spread to all continents. By April 10th, the COVID-19 had caused over 1,521,252 infections with over 92,798 deaths globally (2).

Studies have shown that patients with Diabetes Mellitus (DM) are more susceptible to infectious diseases, including pneumonia (3). Indeed, in human Severe Acute Respiratory Syndrome (SARS) and Middle East Respiratory Syndrome (MERS) patients, diabetic groups were associated with higher mortality than their other counterparts (4, 5). Furthermore, SARS-CoV-2 was found to be a clade from the betacoronaviruses associated with the SARS and MERS-CoV (6).

Previous studies have reported increased severity and mortality on COVID-19 patients with diabetic history (7, 8). However, up to now, only a few studies have focused on the risk factors affecting the prognosis of diabetes patients with COVID-19. Zhu et al. found that a well-controlled blood glucose level (3.9–10.0 mmol/L) was associated with lower mortality as compared to individuals with poorly controlled blood glucose level (>10.0 mmol/L) in patients with COVID-19 and those with pre-existing type 2 Diabetes (9). However, the critical issues concerning treatment principles have remained unclear. Indeed, what risk factors are specific for diabetes patients with COVID-19 infection indicating their clinical progression and outcome, and whether the different anti diabetic therapies potentially affected the clinical course of the COVID-19 patients are still unclear (10). In this regard, this study was aimed at describing the clinical features of diabetes patients with COVID-19 infection and investigating the risk factors influencing the prognosis, especially the impact of different anti-diabetic drugs, through a mono-centered retrospective cohort study.

## Methods

### Study Design

A retrospective cohort study was undertaken on diabetic patients with COVID-19 infection in Central Hospital of Wuhan, a designated medical institution in SARS-CoV-2 infection. We reviewed the medical records of 563 patients with COVID-19 who were admitted in the general ward between January 25th, 2020 and February 14th, 2020, early in this outbreak. After excluding the non-diabetic patients (*n* = 496) as well as those lacking the required important clinical data (*n* = 15), 52 patients were included in the study. The following clinical retrospective data was retrieved from the medical records; demographic features, clinical evaluation, laboratory tests, chest CT, therapies and outcomes. Additionally, composite endpoint for severe clinical events such as admission to Intensive Care Unit (ICU), the need for mechanical ventilation, or death, were followed-up to April 1st 2020. Two physicians (N.L. and M.Z.) independently collected and reviewed the data. The included patients were classified into severe group and non-severe group, based on whether the individuals experienced the severe clinical events. The risk factors associated with the incidence of severe events were analyzed within the study cohort.

This study was approved by the Ethics Committee of Central Hospital of Wuhan and written informed consent was waived due to the rapid spread and the emergency status of this infectious disease.

### Study Definition

COVID-19 was diagnosed based on the criteria of WHO with a confirmed SARS-CoV-2 RNA detection in nasopharyngeal swabs (7). The diagnosis of DM was according to the criteria of the 2020 American Diabetes Association (11). Acute Respiratory Distress Syndrome (ARDS) were diagnosed according to the interim guidance of WHO for COVID-19 (12). Chronic Kidney Disease (CKD) and Acute Renal Injury (AKI) were diagnosed based on the 2012 Kidney Disease Improving Global Outcomes (KDIGO) guideline (13). Acute Myocardial Infarction (AMI) were defined based on the 2017 European Society of Cardiology (ESC) clinical guidelines (14).

### Laboratory Procedures and Chest CT

Laboratory confirmation method for SARS-CoV-2 infection followed the WHO guidelines (1). Nasopharyngeal swabs were tested for SARS-CoV-2 RNA on admission and throughout the clinical course. Laboratory detection of the viral RNA in the swabs was determined by Real-Time reverse-transcriptase Polymerase-Chain-Reaction (RT-PCR) assay as previously described (7). Laboratory tests to evaluate the status of DM included the Fasting Plasma Glucose (FPG), 2 h Post-challenge Glucose (2 h-PG), Hemoglobin A1c(HbA1c), blood Total Cholesterol (TC), High-Density Lipoprotein Cholesterol (HDL-C), Low-Density Lipoprotein Cholesterol (LDL-C) and Triglycerides Levels (TG). Plasma glucose was measured by hexokinase enzymatic method. Blood samples were drawn before breakfast after an overnight fasting of at least 10 h for FPG and 120 min after breakfast for 2 h-PG. HbA1c was performed by using an ultra-high performance liquid chromatograph (UHPLC Nexera X2, Kyoto, Japan). The other routine blood tests performed on admission included; complete blood count, arterial blood gas analysis, serum biochemical tests [renal and liver function, Creatine Kinase (CK), Lactate Dehydrogenase (LDH)], inflammatory biomarkers [high sensitive C Reaction Protein (hsCRP), PCT and Interleukin-6 (IL-6)], cardiac troponin I(cTnI) and D-dimer. To evaluate the COVID-19 pneumonia, chest CT was acquired for all patients following local protocols.

### Statistical Analysis

Continuous and categorical variables were presented as median Interquartile Range (IQR) and *N* (%), respectively. Mann Whitney U-test, χ^2^ test, or Fisher's exact test was applied to compare, where appropriate, the differences between the severe group and the non-severe group. Survival curve on severe event-free survival was presented. Cox proportional-hazard models were used to estimate the Hazard Ratios (HR) and the associated 95% confidence interval (95%CI) for both the univariate and multivariate analysis. In multivariate analysis, White Blood cell (WBC) counts, hs-CRP, CK, LDH, and ESR were excluded for collinearity. Eventually, age, gender, Fasting Plasma Glucose (FPG) levels, cTnI and using of α-GI were included as covariates in the Cox proportional-hazard models of α-GI for severe events, respectively. Interactions with prognostic factors were also examined with the COX proportional-hazards model. All tests were two-sided, and differences with a *p* < 0.05 were considered statistically significant. All analyses were performed using SPSS version 22.0 software, EmpowerStats (R) (X&Y solutions, Inc., Boston, MA) and R (http://www.R-project.org).

## Results

### Demographic and Clinical Characteristics of Severe and Non-severe DM Patients With COVID-19

Five hundred and sixty three patients with COVID-19 were admitted in general ward of Central Hospital of Wuhan between January 25th, 2020 and February 14th, 2020. After excluding the non-diabetic patients (*n* = 496) as well as those lacking the required important clinical data (*n* = 15), 52 patients were included in the final analysis.

According to the level of occurred severe clinical events, the patients were divided into severe group (*n* = 21) and non-severe group (*n* = 31).

Baseline characteristics of the 52 diabetic patients with COVID-19 are present in Table 1. The diabetic patients (9.2%) were from the 563 COVID-19 patients admitted in Central Hospital of Wuhan between January 25th, 2020 and February 14th, 2020. 33 (63.5%) patients were males with median age of 65.50 years (IQR 61.00–72.75). All the patients were diagnosed with type 2 DM and median duration for the disease occurrence on the studied diabetic patients was 10.00 years (IQR 1.25–15.00). In addition to DM, 36 (69.2%) patients had at least one or more other coexisting chronic diseases (Table 1). Thirty four patients (65.4%) and 14 patients (26.9%) had history of essential hypertension and coronary artery disease (CHD), respectively. Furthermore, there was a borderline significant difference in a comorbidity of CHD between two groups (42.9% in severe group vs. 16.1% in non-severe group, *p* = 0.055). It was also found that 22 (42.3%) patients had developed at least one long-term complication of DM prior to the COVID-19 infection. Among the complications, the most common complication was arteriosclerosis (18, 34.6%), followed by diabetic nephropathy (12, 23.1%), then diabetic retinopathy (3, 5.8%) and lastly diabetic foot (1, 1.9%). In the studied cohort, the most common symptoms of the DM patients with COVID-19 on admission were fever 45 (86.5%), dry cough 41 (78.8%), fatigue 32 (61.5%) and dyspnea 22 (42.3%). During the hospitalization time, FPG was monitored in each patient and median value of 8.88 mmol/L (IQR 7.31–11.51) was recorded. HbA1c test was available on 31 patients (59.6%) and median value of 7.2% (IQR 6.5–8.0%) was recorded. Among the patients with HbA1c test, 13 patients (13/31, 41.9%) were in optimal glycemic control with HbA1c at 6–7%, while 18 patients (18/31, 58.1%) were in poor control with HbA1c of above 7% (Table 1). 2 h-PG was tested in 20 patients (38.4%) and median level of 14.80 mmol/L (IQR 11.34–16.38) was recorded. A borderline difference in 2 h-PG was found between two groups (15.20 mmol/L [13.30–18.60] in non-severe group vs. 11.46 mmol/L [10.39–15.65] in severe group, *p* = 0.053). No significant differences occurred in gender, age, disease course of DM, FPG, HbA1c and blood lipid levels between the two groups.

**Table 1:** Demographic and clinical characteristics of severe and non-severe diabetic patients with COVID-19.

	**Total (*n* = 52)**	**Severe (*n* = 21)**	**Non-severe (*n* = 31)**	***p*-value**
Male, *n* (%)	33 (63.5)	14 (66.7)	19 (61.3)	0.774
Age (years)	65.50 (61.00-72.75)	70.00 (62.50–75.50)	65.00 (59.00–71.00)	0.164
BMI (Kg/m^2^)	24.67 (22.35–26.56)	24.34 (22.85–27.03)	24.67 (21.48–26.34)	0.995
Smokers, *n* (%)	15 (28.8)	5 (23.8)	10 (32.3)	1.000
DM history (years)	10.00 (1.25–15.00)	5.00 (1.00–15.00)	10.00 (5.00–15.00)	0.136
Comorbidities, *n* (%)	36 (69.2)	14 (66.7)	22 (71.0)	0.768
EH	34 (65.4)	14 (66.7)	20 (64.5)	1.000
CHD	14 (26.9)	9 (42.9)	5 (16.1)	0.055
CKD	3 (5.8)	2 (9.5)	1 (3.2)	0.558
Complications of DM, *n* (%)	22 (42.3)	11 (52.4)	11 (35.5)	0.263
ASCVD	18 (34.6)	9 (42.9)	9 (29.0)	0.217
Diabetic nephropathy	12 (23.1)	5 (23.8)	7 (22.6)	1.000
Diabetic retinopathy	3 (5.8)	0	3 (9.7)	0.271
Diabetic foot	1 (1.9)	1 (4.8)	0	0.392
**Clinical features**				
Fever, *n* (%)	45 (86.5)	18 (85.7)	27 (87.1)	1.000
Cough, *n* (%)	41 (78.8)	18 (85.7)	23 (74.2)	0.491
Dyspnea, *n* (%)	22 (42.3)	12 (57.1)	10 (32.3)	0.093
Nausea or vomiting, *n* (%)	19 (36.5)	5 (23.8)	14 (45.2)	0.149
Days from onset to Hospital admission	7.00 (6.00–13.25)	7.00 (5.00–17.00)	8.00 (6.00–14.00)	0.587
SBP (mmHg)	128.00 (120.00–142.00)	130.00 (120.00–145.75)	126.00 (120.00–135.00)	0.339
DBP (mmHg)	78.00 (70.00–85.00)	77.50 (70.00–85.00)	78.00 (72.00–85.00)	1.000
HbA1c (%)^**a**^	7.20 (6.50–8.00)	6.75 (6.30–7.63)	7.20 (6.55–9.25)	0.263
HbA1c ≥ 7%, *n* (%)	18 (34.6)	4 (19.0)	14 (45.2)	0.247
FPG (mmol/L, 3.9–6.1^*^)	8.88 (7.31–11.51)	8.20 (6.77–11.00)	9.00 (7.26–11.03)	0.424
2 h-PG (mmol/L, <7.8^*^)^**b**^	14.80 (11.34–16.38)	11.46 (10.39–15.65)	15.20 (13.30–18.60)	0.053
TC (mmol/L; <5.18^*^)	3.71 (3.26–4.59)	3.92 (2.82–4.62)	3.71 (3.29–4.66)	0.733
TG (mmol/L; <1.7^*^)	1.15 (0.88–1.68)	1.26 (0.83–1.71)	1.11 (0.87–1.64)	0.655
HDL-C (mmol/L; >1.04^*^)	0.95 (0.75–1.21)	0.82 (0.58–1.21)	1.01 (0.80–1.26)	0.436
LDL-C (mmol/L; <3.37^*^)	2.14 (1.63–2.80)	1.95 (1.54–2.82)	2.17 (1.76–2.78)	0.524
eGFR (ml/min/1.73 m^2^; 90–120^*^)	116.44 (75.53–140.91)	112.77 (57.86–170.35)	118.08 (77.07–138.02)	0.861
Proteinuria, *n* (%)^c^	9 (17.3)	5 (23.8)	4 (12.9)	0.457

eGFR, estimated glomerular filtration rate; ASCVD, atherosclerotic cardiovascular disease.

Proteinuria defined as the urine protein qualitative test showed positive in the urine routine examination.

^a^ data available in 31 patients;

b data available in 20 patients;

c data available in 29 patients;

* *Normal range*.

### Laboratory Findings

Laboratory results for the admitted diabetic patients with COVID-19 infection are shown in Supplementary Table 1. The blood counts showed higher counts of leukocytes 7.25 × 10^9^/L (IQR 4.87–8.99) and neutrophil counts 6.68 × 10^9^/L (IQR 4.73–8.34) within the severe events diabetic patients with COVID-19 infection as compared to the values of non-severe group (leukocytes 5.26 × 10^9^/L [IQR 3.24–7.18]; neutrophil counts 3.93 × 10^9^/L, [IQR 1.98–6.08]), the difference was statistically significant (*p* < 0.05). Specifically, the neutrophil counts of the severe patients (6.68 × 10^9^/L, IQR 4.73–8.34) was higher than the expected normal range. Lymphopenia was frequently found in the diabetic patients with COVID-19 infection (40, 76.9%), without a different between severe and non-severe diabetic patients with COVID-19. There were numerous abnormal infection-related biomarkers in sera of the diabetic patients, hs-CRP, ESR, PCT, and IL-6 were beyond the normal range. Furthermore, the value of hs-CRP, ESR and PCT in the severe patients were significantly higher than those value in non-severe patients (*p* < 0.05). The difference in IL-6 values between the severe and non-severe patients was borderline significant (*p* = 0.067).

Moderately higher level of cTnI (32.0 pg/ml; IQR 16.80–55.00) was found in patients with severe events as compared to the non-severe patients (20.00 pg/ml, IQR5.38–30.00, *p* = 0.019). Mild increased D-dimer (1.00 μg/L, IQR 0.50–2.40) was also found in the diabetic patients with COVID-19 co-infection, with significantly higher level in the severe patients (1.70 μg/L, IQR 0.70–2.40) as compared to the non-severe patients (0.70 μg/L, IQR 0.30–2.40, *p* < 0.05). Urine routine test was available on 29 patients (55.8%) and 9 (17.3%) patients was positive for proteinuria. The arterial blood gas analysis showed significantly lower PaO_2_ values (61.50 mmHg, IQR 52.8–77.8) in the severe patients than value in the non-severe patients (88.50 mmHg, IQR: 66.80-123.50, *p* < 0.05).

Radiology findings of chest CT for diabetic patients with COVID-19 on admission are similar to the previous report (7), shown in Supplementary Table 1. There was no difference in CT imaging patterns between the severe and the non-severe cases. Bilateral involvement in lungs (47, 90.4%) was more common than unilateral involvement (5, 9.6%). Ground-glass opacities (45, 86.5%) and patchy consolidation (25, 48.1%) were the common CT imaging pattern on the studied patients.

### Treatment, Complications, and Outcome of Severe and Non-severe Diabetic Patients With COVID-19

Treatment and clinical outcome of diabetic patients with COVID-19 are shown in Table 2. In total, 8 (15.4%) patients were put on non-invasive mechanical ventilation and 5 (9.6%) patients had endotracheal intubation and invasive ventilation under progressive hypoxia. 12 (23.1%) of the patients were admitted to ICU as the disease progressed. As of 1st April 2020, 21 (40.4%) of the patients had developed severe clinical events, 23.1% were admitted to ICU, 28.8% experienced life-threatening complications and 8 patients died, with the recorded mortality rate of 15.4%.

**Table 2:** Treatment and clinical outcomes of severe and non-severe diabetic patients with COVID-19.

	**Total** **(*n* = 52)**	**Severe** **(*n* = 21)**	**Non-severe (*n* = 31)**	***p*-value**
**Treatment**				
**Oxygen therapy**, ***n*** **(%)**	23 (44.2)	13 (61.9)	10 (32.3)	0.002
Non-invasive	8 (15.4)	8 (38.1)	0	<0.001
Invasive	5 (9.6)	5 (23.8)	0	<0.001
ECMO	0	0	0	
**Admission to ICU**, ***n*** **(%)**	12 (23.1)	12 (57.1)	0	<0.001
**DM therapy**, ***n*** **(%)**				
Insulin	24 (46.2)	7 (33.3)	17 (54.8)	0.159
α-GI	24 (46.2)	5 (23.8)	19 (61.3)	0.021
Metformin	15 (28.8)	5 (23.8)	10 (32.3)	0.754
DPP4 inhibitor	4 (7.7)	1 (4.8)	3 (9.77)	1.000
Thiazolidinedione	2 (3.8)	2 (9.5)	0	0.149
Sulfonylurea secretagogue	2 (3.8)	0	2 (6.5)	0.514
Non-sulfonylurea secretagogue	2 (3.8)	1 (4.8)	1 (3.2)	1.000
**Complications**, ***n*** **(%)**	15 (28.8)			
ARDS	11 (21.2)	9 (42.9)	2 (6.5)	0.002
Septic shock^**a**^	5 (9.6)	5 (23.8)	0	0.008
Acute kidney injury^b^	4 (7.7)	4 (19.0)	0	0.022
AMI^**c**^	1 (1.9)	1 (4.8)	0	0.404
days form onset to severe event	11.00 (8.50-16.50)	11.00 (8.50-16.50)	NA.	NA.
**Death**	8 (15.4)	8 (38.1)	0	<0.001

ECMO, extracorporeal membrane oxygenation; α-GI, α-Glucosidase inhibitor; DPP4, dipeptidyl peptidase-4; ARDS, acute respiratory distress syndrome; AMI, acute myocardial infarction; NA, not available.

a Three patients had ARDS by co-incidence.

b Two patients had ARDS by co-incidence.

c * One patient had ARDS by co-incidence*.

49 (94.2%) diabetic patients had at least one antiviral agent subscription, such as arbidol (21, 40.4%), ribavirin (29, 51.9%), and ganciclovir (4, 7.7%). Empirical antibiotic was given to most of the patients (47, 90.4%). Systemic corticosteroid (34, 65.4%) and intravenous immunoglobin (20, 38.5%) were also given to the patients. There was no difference in all the medicines provided to the both the severe and the non-severe cases.

Treatment information for DM are also shown in Table 2. 42 (80.8%) patients received hypoglycemic therapy, 8 (15.4%) patients used insulin only, 17 (32.7%) patients used one or more types of oral hypoglycemic drugs while 17 (32.7%) patients used oral hypoglycemic drug combined with insulin. Oral hypoglycemic drugs used included α-glucosidase inhibitors (α-GIs) (24, 46.2%), metformin (15, 28.8%), sulfonylurea secretagogues (2, 3.8%), non-sulfonylurea secretagogues (2, 3.8%), dipeptidyl peptidase 4 (DPP4) inhibitors (4, 7.7%) and thiazolidinediones (2, 3.8%). The proportion of α-GI was significantly higher in the non-severe cases as compared to the severe cases (61.3%vs23.8%, *p* = 0.021).

The most common complication on diabetic patients with COVID-19 was ARDS (11, 21.2%), followed by septic shock (5, 9.6%), then acute kidney injury (4, 7.7%) and lastly AMI (1, 1.9%). From the 52 diabetic patients, 8 (15.4%) patients died within a median time of 10.5 days (IQR 9.0–13.0) from the admission day to the day of death. The cause of death included ARDS (8, 15.4%), followed by septic shock (2, 3.8%) and lastly AMI (1, 1.9%).

### Risk Factors for Developing Severe Event by Univariate and Multivariate Analysis

Results of univariate analysis are shown in Table 3. In univariate analysis, Cox proportional hazards model was used to access the association between the clinical factors and the severe clinical events. The WBC counts, neutrophil counts, LDH levels, creatine kinase levels, hsCRP levels, ESR levels, IL-6 levels and use of α-GI were all significantly associated with severe events (*p* < 0.05). The cTnI levels had a higher association risk of developing severe events with a borderline statistical significance (*p* = 0.097).

**Table 3:** Univariate analysis of the severe events in diabetic patients with COVID-19.

**Variables**	**Univariate analysis**		
	**HR**	**95% CI**	***p*-value**
Gender (female vs. male)	0.86	0.35–2.14	0.748
Age (years)	1.03	0.98–1.08	0.202
DM history (years)	0.96	0.90–1.03	0.289
FPG (mmol/L)	0.98	0.84–1.14	0.761
HbA1c (%)	0.70	0.42–1.16	0.169
BMI (Kg/m^2^)	0.99	0.83–1.18	0.936
HDL-C (mmol/L)	0.43	0.08–2.21	0.31
LDL-C (mmol/L)	0.93	0.48–1.80	0.826
TC (mmol/L)	0.87	0.58–1.30	0.485
TG (mmol/L)	0.97	0.71–1.32	0.829
**Comorbidity**			
EH	0.93	0.37–2.31	0.876
CHD	2.20	0.92–5.23	0.075
CKD	1.94	0.45–8.38	0.373
**DM therapy**			
Insulin	0.50	0.20–1.25	0.138
Metformin	0.73	0.26–2.00	0.537
Thiazolidinedione	5.00	1.14–21.93	0.033
α-GI	0.28	0.10–0.76	0.013
Non-sulfonylurea secretagogue	1.26	0.17–9.41	0.824
DPP4 inhibitors	0.61	0.08–4.59	0.635
**Complications**			
ASCVD	1.30	0.54–3.14	0.558
Diabetic nephropathy	1.11	0.40–3.05	0.845
Diabetic foot	3.47	0.45–26.55	0.231
**Laboratory findings of COVID-19**			
WBC (× 10^9^/L)	1.14	1.01–1.27	0.029
LYM (× 10^9^/L)	1.08	0.75–1.56	0.677
NEU (× 10^9^/L)	1.02	1.00–1.04	0.030
TBil (μmol/L)	1.07	0.98–1.18	0.132
DBil (μmol/L)	1.16	0.97–1.39	0.101
LDH (U/L)	1.00	1.00–1.01	0.006
CK (U/L)	1.01	1.00–1.01	0.048
D-Dimer (μg/L)	1.02	0.97–1.08	0.413
hsCRP (mg/L)	1.01	1–1.02	0.006
PCT (ng/mL)	1.60	0.37–6.96	0.531
cTnI (pg/mL)	1.005	0.999–1.011	0.097
ESR (mm/h)	1.03	1–1.06	0.026
IL-6 (pg/mL)	1.01	1–1.01	0.022

**Figure 1:**
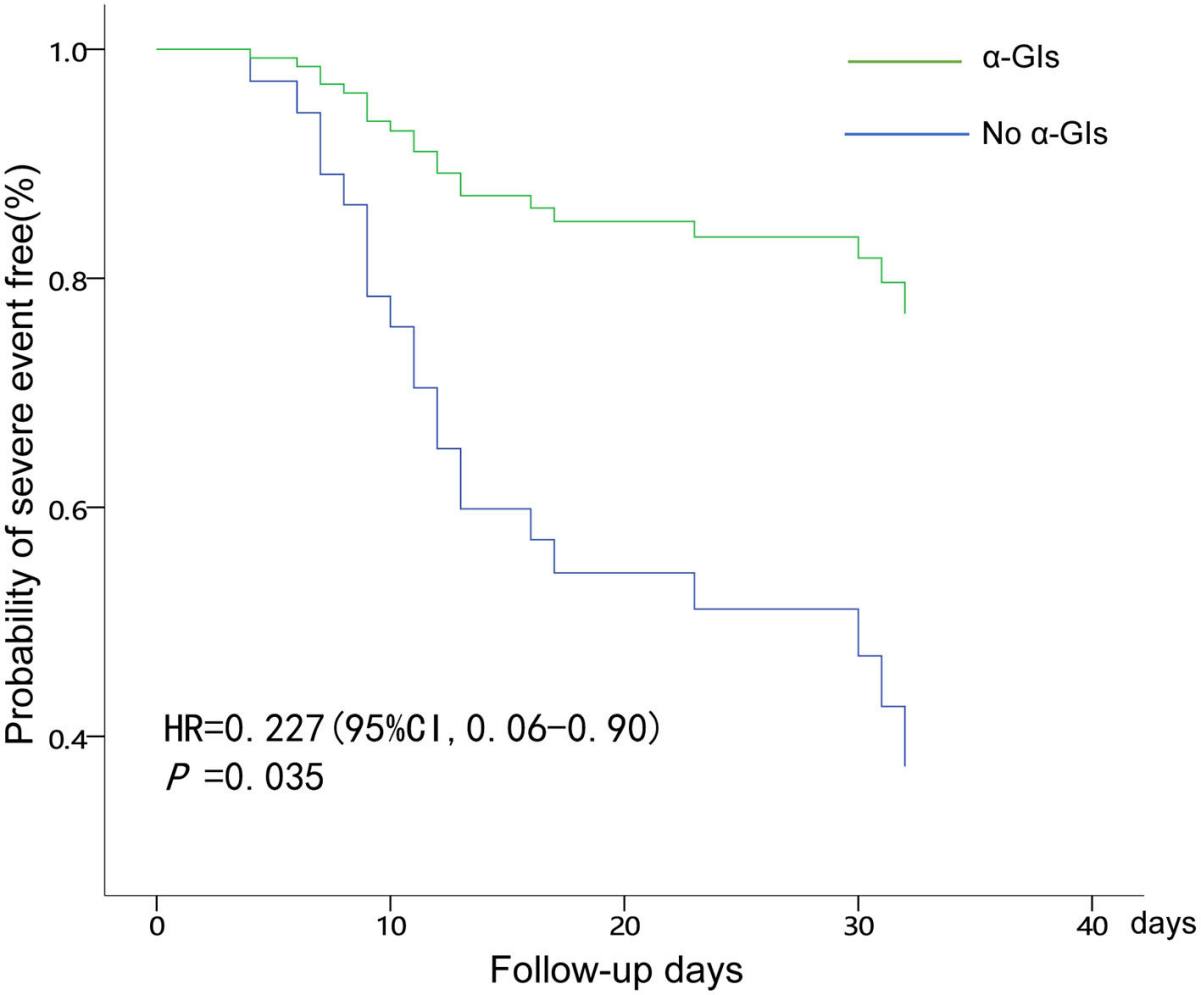
COX regression analysis of administration of α-GIs for developing severe events in diabetes patients with COVID-19.

Results of multivariate analysis on the risk of severe clinical events are shown on Table 4. After being adjusted in regard to age and gender in the multivariate adjusted Cox proportional hazards model, cTnI and α-GI were observed to be meaningful factors associated with the occurrence of severe events. Diabetic patients with COVID-19 who had elevated levels of cTnI had a significant increased risk of developing severe events (HR = 1.01, 95%CI: 1.0–1.01, *p* = 0.048). Diabetic patients with COVID-19 infection who were using α-GIs had 72% lower risk (HR: 0.23; 95% CI: 0.06–0.90; *p* = 0.035) of severe clinical events than those not using α-GIs. The survival curve for severe clinical events based on α-GIs usage are shown in Figure 1.

**Table 4:** Multivariate analysis for the risk of severe events in DM patients with COVID-19 co-infection.

**Clinical factors**	**Multivariate analysis**		
	**HR**	**95% CI**	***p*-value**
Gender	1.930	0.546–6.819	0.307
Age	1.016	0.962–1.074	0.568
FPG	1.005	0.855–1.181	0.953
cTnI	1.007	1.000–1.013	0.048
α-GI	0.227	0.057–0.904	0.035

## Discussion

In the present study, the clinical characteristics of 52 diabetic patients with COVID-19 from a designated hospital in Wuhan, China are described, and the risk factors associated with severe clinical events which were defined as the patients' admission to ICU, the use of mechanical ventilation, or death are investigated. At the follow-up endpoint, 40.4% of the patients developed severe events, 23.1% were admitted to ICU, 28.8% had life-threatening complications, and 8 patients finally died with a recorded mortality rate of 15.4%.

Diabetic patients with COVID-19 infection in the studied cohort were found to partly share similar clinical features with the general population such as; typical symptoms of fever, dry cough, fatigue and dyspnea, along with lymphopenia and high level of infection biomarkers, including hs-CRP and IL-6 (7). However, diabetic patients presented character of higher leucocyte and neutrophil counts, especially in the severe events patients, which were different with the leukopenia frequently found in the general population (7). In addition, for infection-related biomarkers, especially for PCT, the severe events cases had significantly higher levels than those of non-severe cases. Combined with the features of higher leucocyte and neutrophil counts, the higher PCT indicated elevated risks of systemic infection and sepsis in the diabetic patients with COVID-19 infection in the studied cohort.

Furthermore, a recent comparative study had showed that even the diabetic patients with optimal control (HbA1c 6–7%) had an elevated risk of serious infection as compared to patients without diabetes, and the risks rose with increasing HbA1c (15). Majority of the diabetic patients were considered to experience relatively poor glycemic control in the studied cohort. Among the patients with HbA1c test, 58.1% were in poor control with HbA1c of above 7%, which would increase the baseline infection risk as well as the incidence of sepsis.

In the studied cohort, increasing cTnI level was significantly associated with the occurrence of severe clinical events. cTnI is a sensitive biomarker of cardiac injury in variety situation, and was discovered as an important prognostic factor for the patients with COVID-19 infection (16). The mechanism of cardiac injury in COVID-19 was not fully understood, and may be though to be probably related to the presence of underlying CHD, hypoxemia, myocarditis, AMI or septic shock (17). It was noted that 26.9% of the diabetic patients in the studied cohort had a comorbidity of CHD, and the severe events cases had a borderline of higher proportion as compared to the non-severe cases. Endothelial injury is the pathophysiological basis of diabetic vascular disease, and oxidative stress and inflammatory injury play an important role in it. SARS-CoV-2 infects the host through the angiotensin converting enzyme 2 (ACE2) receptor, which is expressed in several organs, including the lung, heart and kidney. ACE2 receptors are also expressed by endothelial cells. Varga et al. (18) indicated the evidence of direct infection of the endothelial cell by the SARS-CoV-2. For the patients with pre-existing endothelial dysfunction and with cardiac dysfunction, which were common in diabetic condition, the cardiac injuries were more severe and the prognosis was worse.

Due to lack of effective therapy for the pandemic, there has been an urgent need for potential agents that can act against the deadly and infectious SARS-CoV-2. The investigation provided interesting information that α-GI could be an independent protector for clinical outcome in diabetic patients with COVID-19 infection. Moreover, the protecting effect is obvious even though only a small portion of α-glucosidase inhibitor is absorbed into circulation (19) Both the SARS-CoV-2 and the Severe Acute Respiratory Syndrome Coronavirus (SARS-CoV) use ACE2 for entry into host cells. Previous studies have found that the N-linked glycan structure of the ACE2 could be altered by glucosidase inhibitors (20). Moreover, inhibition of glucosidases has also been found to impair SARS-CoV and human coronavirus nl63 spike protein-mediated entry into the host cells by altering the N-linked glycan structure (21). Since SARS-CoV-2 also uses ACE2 for entry into host cells, we hypothesized that α-GIs may interfere with SARS-CoV-2 by altering the glycan structure of ACE2 and thus disrupt the entry of the SARS-CoV-2 to the body cells. In addition, inhibition of α-glucosidase has been found to work as antiviral agents for many enveloped RNA viruses, such as the human immunodeficiency virus and hepatitis C virus (22). Both SARS-CoV and SARS-CoV-2 are enveloped RNA viruses which contain N-linked glycans. Treatment with the glucosidase inhibitor N-butyl-deoxynojirimycin inhibits N-glycan processing in SARS-CoV. Therefore, it indicates the potential role of glucosidase inhibitors in the treatment of SARS-CoV-2 infections (23) by disrupting the proper folding and function of the glycoproteins on the SARS-CoV-2 envelope. However, molecular data demonstrating direct antiviral effects of α-glucosidase inhibitors, as well as data on the plasma concentrations of these largely intestinally active and non-absorbed drugs are needed in future studies.

There has been much concern about the pharmacological therapy for diabetic patients with COVID-19. Current recommendations for anti-diabetic drugs for diabetic patients with COVID-19 mainly include maintaining previous medication for the mild cases and changing to insulin for regular and severe cases (10). The present study indicates that α-GIs are independent protective factor for incidence of severe clinical events in COVID-19 infection. Of course, replications in large cohorts are needs to confirm our results.

Even though the study has provided interesting findings, it had some few limitations. First, the study was retrospectively designed from a single center and based on a relatively small sample size, therefore, the roles of the study confounders predicting the severe events might be underestimated. Moreover, it might have unavoidable selection bias as well as heterogeneity. Second, some important confounders were not able to be included in the multivariate analyses, such as the IL-6 and hs-CRP. Although the univariable analyses showed statistically significant association, the study could not include them in the multivariable Cox analysis due to the relatively small sample sizes. Third, since comorbidity of diabetes has been previously reported to lead to more severe and more deadly COVID-19, it would be urgent and to see what risk factors are specific for diabetes patients predicting to the severe course of COVID-19. We only reported and analyzed the outcome and mortality in diabetes patients with COVID-19. The comparisons between diabetes and non-diabetes patients with COVID-19 could reveal more useful information, as would comparisons of less severe cases not included in our study population. Lastly, the present study indicates that using α-GI might have a lower risk of endpoint of severe outcomes, but further randomized control studies with larger sample size are required to ascertain this. Thus, future studies with larger sample sizes and prospective study designs are warranted to further explore the risk factors and potential therapeutic effect of α-GIs in diabetic patients with COVID-19.

In summary, the current study described the detailed clinical features and poor outcome of diabetic patients with COVID-19 in early outbreak of China. The study found increased level of cTnI was a risk factor predicting the incidence of clinical outcome. It is therefore recommend that diabetic patients with COVID-19 infection should have vigorous monitoring of the cTnI. Even more important, we found, α-GIs might have a potential protective effect from severe clinical events for those patients and need to be studied more for clear understanding.

## Data Availability Statement

The raw data supporting the conclusions of this article will be made available by the authors, without undue reservation.

## Ethics Statement

The studies involving human participants were reviewed and approved by the Ethics Committee of Central Hospital of Wuhan. Written informed consent for participation was not required for this study in accordance with the national legislation and the institutional requirements.

## Author Contributions

MZ, NL, NZ, CW, FZ, HM, PB, L-LC, TZ, M-MP, KQ, YW, MY, SX, and JZ implemented the study and collected the data. NZ, CW, FZ, MZ, and NL wrote the manuscript. CW and NZ analyzed the data. All authors participated in the design and interpretation of the studies, analysis of the data and review of the manuscript.

## Conflict of Interest

The authors declare that the research was conducted in the absence of any commercial or financial relationships that could be construed as a potential conflict of interest.
