# Supplementary material for: Risk Factors for Poor Outcomes of Diabetes Patients With COVID-19: A Single-Center, Retrospective Study in Early Outbreak in China
Source: Front Endocrinol (Lausanne). 2020 Sep 24;11:571037. doi: 10.3389/fendo.2020.571037 (PMC7543084; doi:10.3389/fendo.2020.571037)
Supplement: Supplementary file 1 [file Table_1.DOCX]

**Supplementary Table 1**  **Laboratory tests and radiology findings of severe and non-severe diabetic patients with COVID-19.**

|  | Total(n=52) | Severe (n=21) | Non-Severe (n=31) | *p* value |
| --- | --- | --- | --- | --- |
| **Blood Routine** |  |  |  |  |
| WBC (×10^9^/L; 3.5-9.5^*^) | 6.02(4.54-8.30) | 7.25(4.87-8.99) | 5.26(3.24-7.18) | 0.023 |
| LYM (×10^9^/ L; 1.1-3.2^*^) | 0.90 (0.30-7.40) | 0.68(0.54-0.96) | 0.91(0.68-1.14) | 0.186 |
| NEU (×10^9^/ L; 1.8-6.3^*^) | 4.82 (3.22-7.35) | 6.68(4.73-8.34) | 3.93(1.98-6.08) | 0.001 |
| Hb (g/L; 130.0-175.0^*^) | 128.0(114.00-138.00) | 128.0(107.30-141.00) | 127.0(120.00-134.00) | 0.756 |
| PLT (×10^9^/L; 125.0-350.0^*^) | 165.0(119.00-200.00) | 169.5(118.30-215.50) | 165.0(123.00-189.00) | 0.966 |
| **Blood Biochemistry** |  |  |  |  |
| TBil (μmol/L; 0.0-26.0^*^) | 9.20(7.30-13.50) | 10.60(7.60-15.80) | 9.10(7.00-11.50) | 0.168 |
| DBil (μmol/L; 0.0-8.0^*^) | 4.10(2.60-5.40) | 4.90(3.40-5.80) | 3.50(2.20-4.60) | 0.023 |
| ALT (U/L; 9.0 -50.0^*^) | 19.30(12.10-32.10) | 20.10(14.00-29.00) | 19.30(12.00-36.30) | 0.875 |
| AST (U/L; 15.0-40.0^*^) | 28.00(19.00-39.80) | 27.60(20.50-39.10) | 28.00(18.00-44.80) | 0.875 |
| BUN (mmol/L; 3.6-9.5^*^) | 5.60(4.20-7.40) | 6.50(5.20-10.90) | 5.30(4.10-6.10) | 0.051 |
| Cr (mmol/L; 57.0-111.0^*^) | 66.00(54.90-84.20) | 68.20(48.50-106.80) | 63.80(55.50-82.00) | 0.857 |
| LDH (U/L; 120.0-250.0^*^) | 253.50(194.30-347.50) | 302.00(210.00-458.00) | 232.00(191.00-317.00) | 0.100 |
| **Infection-related biomarkers** |  |  |  |  |
| hsCRP (mg/L; 0.0-5.0^*^) | 8.80(4.70-25.40) | 12.20(7.90-54.30) | 5.60(3.50-13.00) | 0.009 |
| ESR (mm/h; 0.0-15.0^*^) | 42.00(24.00-72.00) | 72.00(39.00-80.00) | 34.00(17.00-56.50) | 0.020 |
| PCT (ng/ml; 0.0-0.50^*^) | 0.10(0.10-0.20) | 0.10(0.10-0.30) | 0.10(0.10-0.20) | 0.005 |
| IL-6 (pg/ml; 0.0- 7.0^*^) | 20.80(6.10-43.10) | 34.60(19.30-69.00) | 15.70(4.10-41.30) | 0.067 |
| **Other tests** |  |  |  |  |
| D-dimer (μg/L; 0.0-0.5^*^) | 1.00(0.50-2.40) | 1.70(0.70-2.40) | 0.70(0.30-2.40) | 0.037 |
| cTnI (pg/ml; 0.0-34.2^*^) | 22.00(10.00-35.00) | 32.00(16.80-55.00) | 20.00(5.38-30.00) | 0.019 |
| **Arterial Blood Gas Analysis** |  |  |  |  |
| pH (7.35-7.45^*^) | 7.40(7.40-7.50) | 7.40(7.40-7.50) | 7.50(7.40-7.50) | 0.638 |
| PaO_2_ (mmHg;80-100^*^) | 74.00(56.00-101.00) | 61.50(52.80-77.80) | 88.50(66.80-123.50) | 0.044 |
| PaCO_2_ (mmHg;35.0-45.0^*^) | 36.00(33.50-38.00) | 35.00(30.00-38.00) | 36.00(34.00-38.50) | 0.320 |
| **CT scan on admission, n (%)** |  |  |  |  |
| Bilateral involved | 47(90.4) | 20(95.2) | 27(87.1) |  |
| Unilateral involved | 5(9.6) | 1(4.8) | 4(12.9) | 0.637 |
| Ground-glass opacities | 45(86.5) | 18(85.7) | 27(87.1) | 0.220 |
| Patchy consolidation | 25(48.1) | 11(52.4) | 14(45.2) | 0.778 |
| Interstitial abnormalities | 5(9.6) | 2(9.5) | 3(9.7) | 1.000 |

WBC, white blood cell; LYM, lymphocyte; NEU, neutrophil; Hb, Hemoglobin; PLT, platelet; TBil, total bilirubin; DBil, Direct Bilirubin; ALT, alanine aminotransferase; AST, aspartate aminotransferase; LDH, lactate dehydrogenase; CK, creatine kinase; BUN, urea nitrogen; Cr, creatinine; PCT, procalcitonin; cTnI, cardiac troponin I; IL-6, Interleukin-6; PaCO_2,_ Partial Pressure of Carbon Dioxide; PaO_2_, Partial Pressure of Oxygen.

* Normal range.
